# Rapid adaptation in phoretic mite development time

**DOI:** 10.1038/s41598-018-34798-6

**Published:** 2018-11-07

**Authors:** Petra Schedwill, Adrian M. Geiler, Volker Nehring

**Affiliations:** Evolutionary Biology & Ecology, Institute of Biology I, University of Freiburg, Hauptstraße 1, 79104 Freiburg, Germany

## Abstract

Strong ecological selection can erode genetic variation and render populations unable to deal with changes in ecological conditions. In the adaptation of the phoretic mite *Poecilochirus carabi* to its host, the burying beetle *Nicrophorus vespilloides*, the timing of reproduction is crucial. Safe mite development is only possible during the beetles’ brood care; mites that develop too slowly will have virtually zero fitness. If the strong specialisation in development time leaves no room for standing genetic variation to remain, changes in beetle brood care are disastrous. Beetle brood care depends on temperature and is thus vulnerable to changing climate. Accidental host switches to another beetle species with shorter brood care would also have negative effects on the mites. Only sufficient standing genetic variation could allow mismatched mite lines to survive and adapt. To test whether such rapid adaptation is possible in principle, we artificially selected on mite generation time. We were able to speed up, but not to slow down, mite development. We conclude that there is enough standing genetic variation in development time to allow *P. carabi* to quickly adapt to new host species or climate conditions, which could potentially lead to the evolution of new mite species.

## Introduction

Life history traits are subject to strong selection due to their direct link to fitness, which often reduces genetic variation^1–4^. As adaptation in response to selection is only possible when there is genetic variation to be drawn upon^5^, populations may fail to adapt to new environments that require different life histories. This limits the evolutionary potential of populations, e.g. the ability for range expansion, or to cope with altered environment, in particular when the novel ecological conditions are extreme^6,7^. While life history traits may not typically come to mind when thinking about adaptation to changing environment, they may be important ecologically selected traits in some symbiotic species. Whenever life history traits evolve to match those of another symbiont species, deviation from the optimum can lead to symbiont mismatch and zero fitness. In these cases, the viable range of the life history trait may be particularly small and genetic variation may quickly erode. This type of genostasis could then prevent rapid adaptation upon host switches. We investigate a phoretic mite species whose juvenile development is adapted to the brood care behaviour of its host beetles. Crucially, when mite development takes too long, the mites might miss the parental beetles that are the best vectors for mite dispersal, leaving the mites with zero or very low fitness.

Mites of the *Poecilochirus carabi* species complex^8^ disperse in the deuteronymphal state on burying beetles (*Nicrophorus*), which reproduce on small vertebrate carcasses^8,9^. The fitness effects of mites on beetles are not entirely clear. Under specific circumstances, the beetles may benefit from carrying mites when mites can keep beetle competitors, such as flies, at bay^10,11^. In simple laboratory experiments, however, beetle fitness suffers in particular from high mite densities^12,13^. After mites arrive at a carcass, they leave their host beetle and within few hours moult into adult females and males^14–16^. Adult mites mate and females lay their eggs into the soil surrounding the carcass^15,17–19^. Hatching larvae moult into protonymphs before moulting into deuteronymphs, the last juvenile instar and only phoretic stage. While the mites develop, the beetles reproduce and take care of their young^20^. Beetle brood care usually assures that the parental beetles are still at the carcass when mite development is complete, so that the new generation of deuteronymphs can attach to the parental beetles and disperse with them^18,19,21^. If the parental beetles have already left before the deuteronymphs develop, the mites have to wait for the beetle offspring to disperse. Beetle offspring will not complete their development for another three weeks or longer^8,10,18,21,22^ and the pupae are exposed to predators and parasites^18,23^. A development that is too slow will thus severely reduce mite fitness.

There are many *Nicrophorus* species around the world that differ in the duration of their brood care^8,22,24,25^. Within the *P. carabi* species complex, the different mite species are associated with different beetle species. Mites are specialised on one main host species, and one crucial adaptation is that mite development time matches beetle brood care duration^8,13,22^. In Europe, for example, females of *Nicrophorus vespillo* provide brood care for about 16 days, and females of *N. vespilloides* for only 11 days^13,26^. Correspondingly, the generation time of *P. carabi* (associated with *N. vespilloides*) is shorter than that of *P. necrophori* (associated with *N. vespillo*)^13^.

Different beetle species interact on feeding carcasses that are too large for reproduction and in direct contests over reproduction resources^15^. Mites dismount the beetles in these cases and may lose contact to their carrier. If they subsequently associate with another *Nicrophorus* species, fitness may be greatly reduced if the mite generation time is longer than the new host’s brood care. In these cases, the mismatch will pose strong selection pressure on the mites. If variation in mite generation time is limited, mite lines exposed to host mismatch may simply become extinct. If there is enough genetic variation for selection to act upon, however, mismatched mite lines may rapidly adapt and specialise on the new host species. The combination of a relatively high likelihood of host switches with strong host-specific selection on life history traits may be a significant generator of mite biodiversity^27^. While there are only two species described within the European *P. carabi* species complex^9^, the global diversity is likely much higher. There are two North American populations that are reproductively isolated from each other^22^, and preliminary genetic analyses indicate that there may be five or more different species world-wide (Schedwill & Nehring unpublished data).

Here, we test in an artificial selection experiment whether there is enough standing genetic variation in *P. carabi* populations to allow for a rapid adaptation in generation time, for example following a host switch. We selected bidirectionally on shorter and longer generations in the mites. After four generations of selection, we measured the timing of mite development and found that mites could indeed quickly adapt to hosts with shorter brood care duration.

## Methods

**Figure 1:**
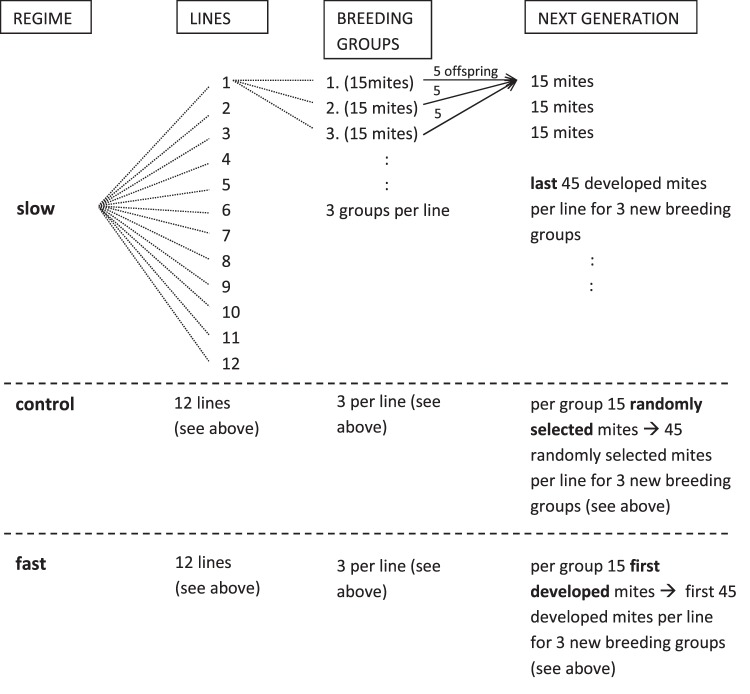
Schematic design of one generation of selection in three regimes. Each of the three selection regimes consisted of twelve replicate lines. Each line again comprised three breeding groups of 15 mites each. In every new generation, each of the three breeding groups was created by combining five offspring per each of the previous generation’s three breeding groups.

**Figure 2:**
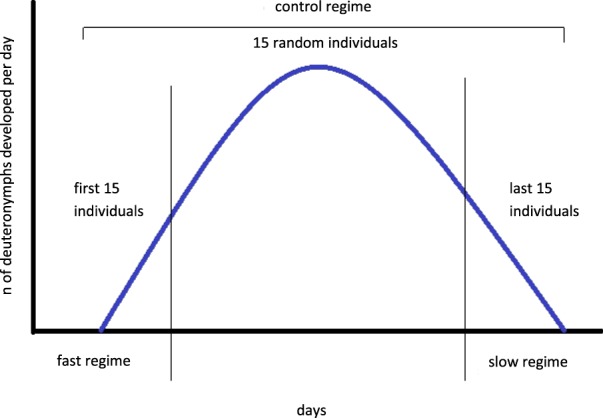
The mode of selection within mite breeding groups. For the control regime, we waited until all deuteronymphs had moulted and selected 15 individuals at random to produce the next generation. For the fast and slow regimes, we selected the 15 first or last deuteronymphs, respectively. Three breeding groups constitute one replicate line.

We exposed *Poecilochirus carabi* mites to three different regimes: two experimental regimes, one selecting for short and one for long generation times, and one control regime without selection (Figs. 1 and 2). Each regime was replicated in twelve independent lines (Fig. 1). All 36 lines originated from the same stock population. Artificial selection was planned to run for ten generations with detailed examination of each line’s generation time (reproduction in pairs instead of groups) after four and ten generations. However, breeding success declined around the seventh generation for unknown reasons, so that several lines went extinct or through strong bottlenecks. An analysis of any effects of selection after this point was thus unreliable and we will focus here on the effects after four generations of selection.

### Mite collection, housing and breeding

*Poecilochirus carabi* were captured in September and October of 2014 with their hosts *Nicrophorus vespilloides* in a south German forest near Freiburg im Breisgau. Mites were bred without beetles at a temperature of 20 °C and in the dark, in plastic boxes of 10 × 10 cm, filled with a 1.5 cm layer of moist peat. We provided a piece of fresh liver (2 × 2 × 1 cm) as a reproduction resource, placed on a small Petri dish lined with paper cloth. Developed deuteronymphs were collected by adding a beetle into the breeding box. The mites would then climb onto the beetles so that we could remove them together. Afterwards, mites were kept on beetles at 20 °C and a 16/8 light-dark cycle in peat-filled plastic boxes for at least three days before starting each new generation. To remove mites from the beetles, we anesthetised both with CO_2_ so that the mites would fall off. Mites were bred in groups of 15 deuteronymphs (“breeding groups”).

### Stock population

Before we began selection, we created a large stock population of field-caught mites that we randomly interbred for four generations to allow for potential adaptations to the laboratory environment and to decrease genetic linkage^28^. For each generation, all deuteronymphs, after resting on beetles for 2–3 weeks, were pooled in a single box and then randomly assigned to breeding groups of 15 individuals each. The number of breeding groups increased with proceeding generations, starting with nine groups in the first and 36 breeding groups in the fourth generation.

### Selection

We selected on generation time, the duration from mites receiving a resource (liver), which triggers the adult moult, until the mites of the next generation moult into deuteronymphs. We created three regimes composed of twelve replicate lines each (Fig. 1). In the fast regime, we used for further reproduction only the 45 individuals of each line (15 per breeding group) that moulted into deuteronymphs first (Fig. 2). In the slow regime, the 45 mites that developed last in each replicate line were used for further reproduction. Finally, we created a control regime by collecting all developing deuteronymphs and then randomly selecting 45 mites from each line, as we did when creating the stock population.

In each generation, the 45 mites per line were split up into three breeding groups of 15 mites each. For each breeding group, five mites from each of the previous generation’s breeding groups were combined (Fig. 1). This way we controlled outbreeding to increase the effective population size within the lines^29^.

The breeding groups were checked daily to note which instars were present and to count the number of developed deuteronymphs. Breeding conditions were the same as for the stock population and mites were removed from the dark climate chambers only for the daily checks, which we typically conducted in the morning. First deuteronymphs appeared usually between 5–13 days (median 7) after setting up the breeding groups. From then on, we collected the mites every day by adding one to two beetles to the breeding boxes for 5–10 minutes, as described above. We gave all breeding groups the opportunity to produce deuteronymphs for at least 14 days and stopped them after 2–3 days on which only few new deuteronymphs would appear per day, which resulted in a maximum of 18 days. We kept the deuteronymphs that were collected on different days and from different breeding groups separately at 20 °C and a 16/8 light-dark cycle. We transferred all mites to beetles for three days before we started a new generation, at which time the fast and slow deuteronymphs were of similar age (3–5 days, depending on how many days we needed to harvest mites to reach 45 individuals per line). Control mites varied in age since the earlier developing mites were naturally 4–11 (median 9) days older than the later developing mites. We analysed developmental speed for the first deuteronymph (quasipoisson error family) as well as for median generation time (Gaussian errors) using linear models with regime and line as independent variables. All statistical analysis were conducted in R (version 3.4.4).

### Measuring development time in mite pairs

When mites were bred in breeding groups of 15 individuals during the selection, the exact timing of mating and development was difficult to record because it was easy to miss individuals, and because due to the scale of the project we had a relatively loose recording schedule. To obtain more exact data, we bred mites of the fourth generation in pairs instead of groups. We collected deuteronymphs from this generation and triggered the adult moult by setting them up in groups of 15 on a piece of liver. After 12–18 hours on average, the deuteronymphs could typically be isolated and would still moult even without the presence of a mating partner. Per line, 7–15 pairs of adult male and female were then joined on a little piece of liver in small rectangular boxes (5 × 3 × 1.5 cm) with a 0.5 cm layer of gypsum on the bottom (exceptions: line no. 5 slow regime (two pairs), line no. 9 control regime (no pairs)). We waited for the pairs to copulate and then checked the boxes twice a day at exact times (a ca. 2 h window) and recorded the first appearance of each developmental stage (egg, larva, protonymph, deuteronymph). Once individuals moulted into deuteronymphs, we removed them from the breeding box by holding a beetle above the box, so that the deuteronymphs climbed onto it. To estimate developmental time, we recorded the middle between the check in which a freshly moulted deuteronymph was found and the previous check. For the analysis, we only used pairs from which more than two offspring developed to deuteronymphs. We analysed offspring number (quasipoisson error family) as well as the developmental speed (Gaussian errors) using linear models with regime and line as independent variables.

### Morphological response to selection

To test whether our selection affected the morphology of the deuteronymphs, either incidentally or through a trade-off between development time and growth, we measured the width and length of the podonotal and opisthonotal shields of fourth-generation deuteronymphs that were not used to form the next generation (measured at 39.4 x magnification, pictures were taken and measured with Leica Application Suite (LAS, Version 4.9.0)). We used these measurements because they correlate with body size, are not affected by storage in ethanol, and have been found to differ between the two species *Poecilochirus carabi* and *Poecilochirus necrophori*, which also differ in generation time^9^. Ten individuals per line were measured.

We approximated the area of the shields by multiplying width and length and also fed the four individual measurements into a principal component analysis. Approximated shield areas were analysed using linear models with regime and line as independent variables (Gaussian errors). The principal component analysis was plotted using R-package *factoextra* (v1.0.5)^30^ and principal components were analysed using linear models with Gaussian errors.

## Results

### Selection

**Figure 3:**
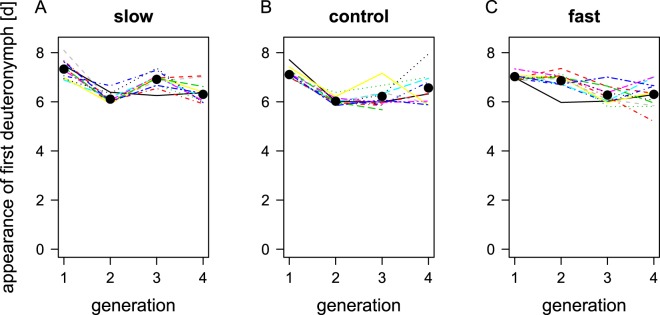
Time from setting up breeding groups until the emergence of the first deuteronymph over four generations of artificial selection for the three selection regimes slow (**A**), control (**B**), and fast (**C**). Each regime consists of twelve lines (different line types and colours). Each curve plotted here is the mean of three breeding groups. Dots show mean per regime, calculated over the twelve lines.

The first deuteronymphs appeared 5–13 days (median 7 days, IQ 6–7) after breeding groups were set up. The generation time of the quickest deuteronymphs (glm with quasipoisson errors, Chisq-Test, ANOVA, p < 0.001) and the median generation time over all deuteronymphs of a breeding group (Gaussian glm, ANOVA, F_1,431_ = 8.2, p < 0.01) decreased over the four generations in a similar way for all three selection regimes (interaction generation x regime p = 0.13 and p = 0.25, respectively; Fig. 3). Overall, the first deuteronymphs developed latest in the lines of the slow regime (glm with quasipoisson errors, Chisq-Test, ANOVA, p < 0.05), but the median generation time of the three regimes did not differ (ANOVA, F_2,431_ = 1.6, p = 0.21). Within regimes, the effect size of the factor generation was at least twice as high in the fast regime (28% deviance explained) than in the other two regimes (slow 14%, control 5% of deviance explained). This suggests that generation time fluctuates more randomly in the slow and control regimes (where we also could observe increases in generation time from one generation to the next) than in the fast regime, where the change was more consistent and likely caused by the experimental selection regime. The per-generation data suffer from loose recording intervals and individuals potentially hiding in the peat.

The average selection differential per generation (average generation time per breeding group minus average generation time of deuteronymphs used to initiate the next generation) was s = −2.36 (sdev 0.29) for fast lines and s = 3.01 (sdev 0.23) for slow lines (t.test p < 0.001).

### Development when bred in pairs

When we bred the offspring of the fourth generation in pairs, there was no difference in offspring number between the regimes (glm with quasipoisson errors, Chisq-Test, p = 0.38). Within the regimes, the different lines varied in offspring number (p < 0.001).

**Figure 4:**
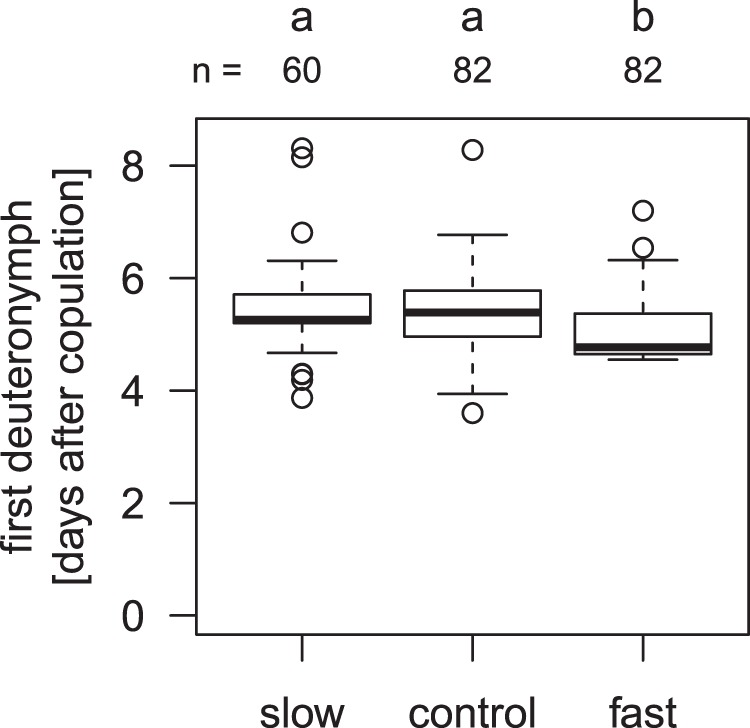
Generation time in days from the first copulation of the parents until the first individual moulted into a deuteronymph. Artificial selection could shorten but not prolong generation time. Boxplots depict median (thick line), interquartile range (box), minimum and maximum. Numbers are sample sizes; groups with identical letters do not differ from each other in a Tukey post-hoc test, p < 0.05.

The overall generation time of the mites, from the copulation of the parent pair until the appearance of the first deuteronymph, was affected by the selection regime we applied (ANOVA, F_2,223_ = 7.80, p < 0.001). Interestingly, the lines within the regimes also differed in their generation time (ANOVA, F_32,223_ = 2.94, p < 0.001, Supplementary Fig. S1). The first deuteronymph developed earlier in the fast regime than in both the slow regime and the control group (Tukey post-hoc test, p < 0.05 in both cases, Fig. 4). In the slow regime, the first deuteronymph appeared after a median of 5.4 days, in the control regime after 5.3 days, and in the fast regime after 4.8 days.

A Tukey test for honest significant differences, for each regime separately, singled out two lines in particular (slow 4 and fast 1) that appear not to have reacted to selection (Supplementary Fig. S1). In these lines, the founding subpopulations may have lacked the genetic variation necessary for rapid adaptation. The qualitative effect of the regime does not change when we re-run the analysis excluding these two conspicuous lines (ANOVA (factor regime), F_2,206_ = 14.36, p < 0.001).

The median time for deuteronymph generation time (time until half of the deuteronymphs were developed) similarly differed among the three regimes (ANOVA, F_2,223_ = 3.16, p < 0.05, Supplementary Fig. S2), but not between lines (F_32,223_ = 1.47, p = 0.06). It was shorter in the fast regime than in the control group (Tukey post-hoc test, p < 0.05), but the slow regime did not differ from the two other regimes (Tukey post-hoc test, p > 0.2 in both comparisons). The time between the parents’ copulation and the moult of the the last deuteronymph did not differ between the three regimes or among lines (ANOVA, F_2,223_ = 0.69 and F_32,223_ = 1.09, p > 0.35 in both cases).

**Figure 5:**
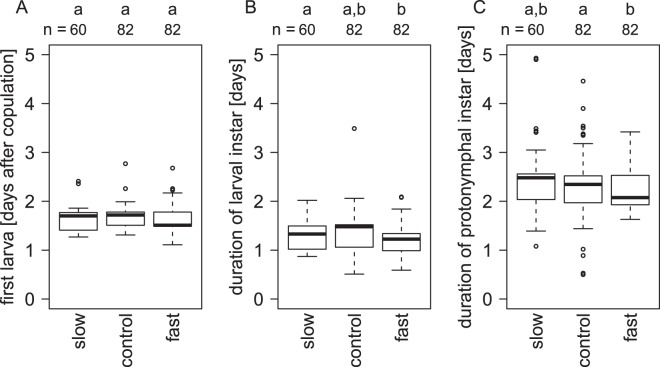
Appearance and duration of larval and protonymphal instars during mite development. The first larvae (**A**) hatched at similar times in all three selection regimes. The duration of the larval (**B**) and the protonymphal instar (**C**) depended on the selection regime. Numbers are sample sizes; groups with identical letters do not differ from each other in a Tukey post-hoc test, p < 0.05.

To understand how the variation in development among the regimes is produced, we analysed the duration of the different instars: Eggs were generally laid in the first 24 hours after copulation, often already in the first 12 hours. The first larva appeared after a median of 1.5 to 1.7 days after copulation (Fig. 5A). The timing of larval hatching did not differ between the selection regimes (ANOVA, F_2,223_ = 0.95, p = 0.39, Fig. 5A), but between lines (ANOVA, F_32,223_ = 2.36, p < 0.001). The duration of the larval instar however, from the first larva hatching to the first moult into a protonymph, was shorter in the fast regime than in the slow regime (Tukey post-hoc test, p < 0.05, Fig. 5B) and the duration of the next stage, the protonymphal instar, was shorter in the fast regime than in the control regime, without a difference to the slow regime (Tukey post-hoc test, p < 0.05, Fig. 5C).

When we compared among the lines of the fast selection regime, we found differences between the lines in the duration of both the larval (ANOVA, F_11,81_ = 7.97, p < 0.001) and the protonymphal instar (ANOVA, F_11,81_ = 10.98, p = 0.03). In the larval instar some lines were particularly quick (Supplementary Fig. S3). While the duration of all instars was generally positively correlated with the overall generation time, positive correlations among stages were weak or absent (Table 1). This might indicate that individual selection lines varied in how they achieved a reduction in overall generation time.

**Table 1:** Correlation coefficients for the duration of the different juvenile instars and the overall generation time for the fast selection regime, calculated from line averages.

	Larva	Protonymph	Overall generation time
Egg laying + egg development	−0.14	0.61*	0.64*
Larva		0.11	0.54
Protonymph			0.85***

Stars indicate significance in a Pearson’s product-moment correlation (*p < 0.05; ***p < 0.001); only values printed in bold are significant after Bonferroni-Holm correction for multiple testing.

### Morphological differences between selection regimes

**Figure 6:**
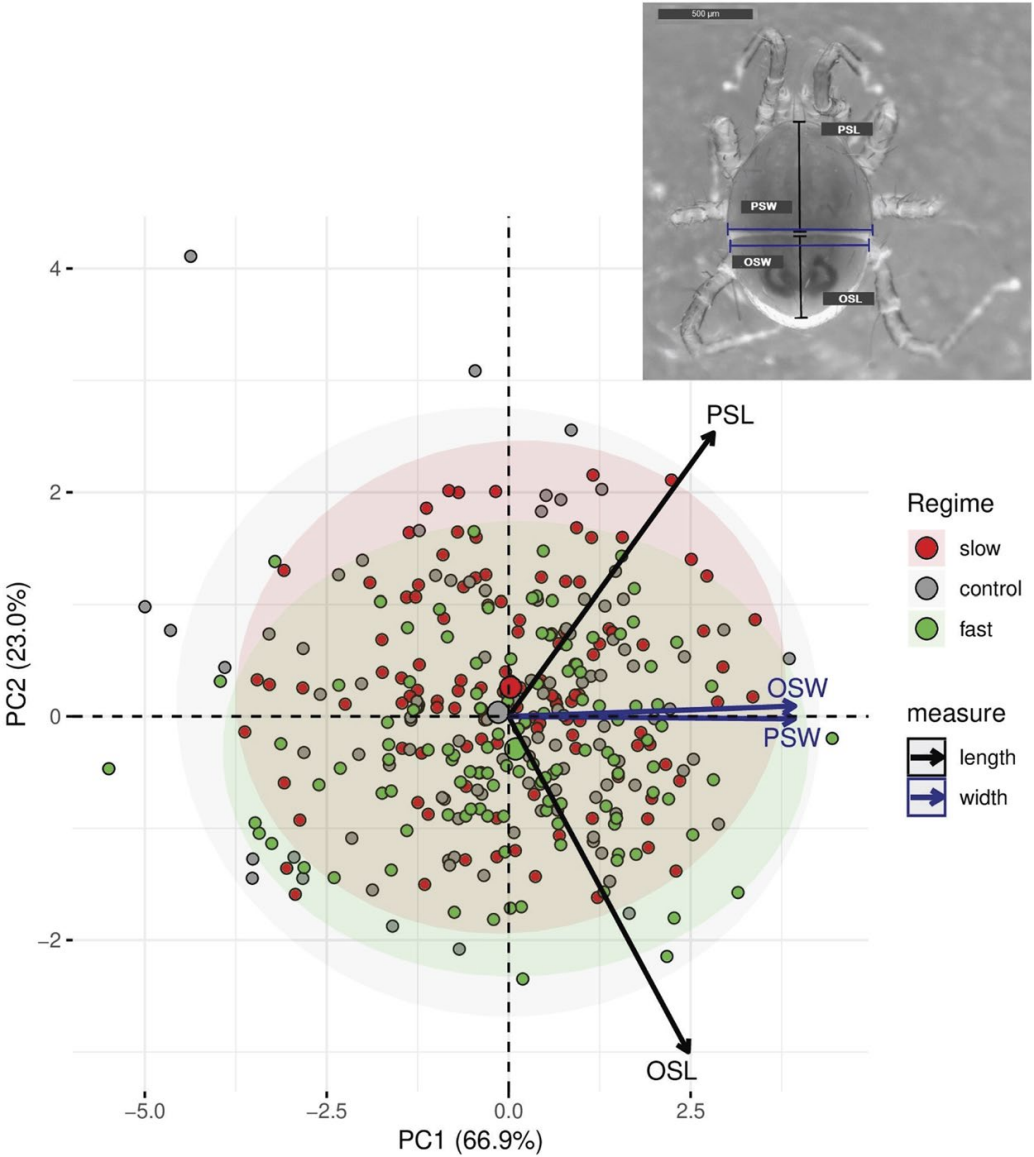
Principal component analysis of the length and width of the podonotal (PSL and PSW) and opisthonotal shield (OSL and OSW) of deuteronymphs after four generations of selection. The three selection regimes are shown in different colours (grey = control, green = fast, red = slow), arrows indicate correlation of PCs with the four measured characters, and circles are 95% confidence intervals. The three large circles indicate mean values of the regimes. There is one outlier in the control regime, which represents an individual with a short opisthonotal shield (top left). Otherwise, this individual had no abnormalities in body shape and structure. When we removed this sample from the analysis, the results did not change. Insert in the top right corner: A *Poecilochirus carabi* deuteronymph: The blue and black lines indicate how width and length of the podonotal (PSW, PSL) and the opisthonotal shield (OSW, OSL) were measured.

We found the approximated area of both the podonotal (F_31,339_ = 2.9, p < 0.001) and the opisthonotal shield (F_31,339_ = 3.4, p < 0.001) to vary among selection lines. While the selection regime had an effect on the size of the opisthonotal shield (F_2,339_ = 6.4, p < 0.01), it had no effect on the podonotal shield (F_2,339_ = 1.7, p = 0.18). Mites of the fast selection regime had a larger approximated area of the opisthonotal shield than slow selected mites and the control regime (Tukey post-hoc test, p < 0.01). In a principal component analysis, the first two components explained 89.92% of the total variance in the four individual measures. The first component separated individuals according to size, while the second component separated individuals with a long opisthonotal shield from those with a long podonotal shield (Fig. 6). While the selection regime did not affect the placement of individuals on the first (overall size-related) component (ANOVA, F_2,337_ = 0.61, p = 0.54), the second component affects the relative shape of podonotal and opisthonotal shields: Individuals from fast selection lines had longer opisthonotal shields than those from the other two regimes, which had an elongated podonotal shield (F_2,339_ = 10.6, p < 0.001; Tukey post-hoc test, control-fast p < 0.05, control-slow p = 0.18, fast-slow p < 0.001). The variance of the control group differed from the two selection regimes for the second component of the principal component analysis (Bartlett Test, p < 0.05). Both selection regimes, fast and slow, varied less than the control group.

## Discussion

We examined the potential for rapid adaptation in phoretic mite generation time, a life history trait that is also crucial for host specialisation. When compared across species, generation time generally matches the duration of host brood care. Accidental host switches can lead to mismatches that cause steep fitness declines. However, rapid adaptation to the changed conditions would allow the mites to leave this fitness valley by adjusting to the environment provided by the novel host. By experimentally selecting on generation time of *Poecilochirus carabi*, we could shorten overall mite generation time, with quicker development in the larval and protonymphal instars. This indicates that there is enough standing variation in this life history trait to rapidly adapt when conditions change.

### Decreased developmental time in fast selection lines

Overall, quicker development in the selected lines was due to shorter larval and protonymphal instars. However, the durations of the different instars were not necessarily correlated, indicating that lines varied in how they responded to selection, e.g. because some shortened the larval and others the protonymphal instar. Interestingly, selection reduced the time until the first deuteronymph moulted and the median generation time over all offspring, but not the duration until the last deuteronymph moulted. We selected on a complex trait that depends not only on the offspring’s development but also on adult moult, mating, and egg laying. Female egg laying takes place over a few days^15,18^ and there may be limits to how much it can be condensed. Females begin laying eggs at high rates and later egg laying trickles away^19^. It is therefore possible that individuals that moulted into deuteronymphs later in the fast selection lines had also increased developmental speed, but hatched from late-laid eggs.

### No effect of selection for longer generations

Selection for longer generation time had no effect in our experiment. For the slow regime, we used the mites we collected last from the breeding boxes. It is possible that at least some of these might actually have completed their development earlier but remained hidden in the peat for some days. Further, the last deuteronymphs emerging may actually have developed quickly but simply stem from late-laid eggs (see above). Hence, the selection in the slow regime was likely not as strong or consistent as that under the fast regime.

### Potential laboratory artefacts

In addition to the weak selection for longer generation time, some other factors might have limited the selection effects in our experiment. With 45 individuals per line per generation, the populations were rather small, which comes with the risk of inbreeding, and which might have contributed to the breakdown of some lines in later generations. This effect would be particularly important because life history traits appear to be more strongly affected than others by inbreeding^31^. However, since population size did not fluctuate and there is no reason to assume any deviation from an even sex ratio, the effective population size in our experiments was likely close to the census population size of N_c_ = 45 per line^28,32^. To ensure a large effective population size, we further actively outbred individuals within lines by controlling individuals to evenly “migrate” between these sub-populations in every generation^29^ (Fig. 1). Effects of inbreeding are unlikely to occur within fewer than ten generations whenever the effective population size is over “a few dozen”^28^, so that strong effects of inbreeding within the four generations analysed here seem unlikely.

Generation time measured from breeding groups seemed to decrease in all selection regimes in parallel over four generations (Fig. 3). This would conflict with our main result that the fast lines developed quicker than the control and slow lines after four generations, obtained by measuring development in pairs (Fig. 4). However, if we consider that measuring development in breeding groups was much less accurate than it was in the fourth generation’s pairs (fewer checks, less consistent timing of the checks, difficulties of locating all moulted deuteronymphs in large breeding groups), the seeming contradiction between measurements from breeding groups and pairs might be more due to experimental and statistical error than to real effects in the breeding groups data set.

Further, a close look at the data from four generations of breeding groups indicate that the reduction in generation time might not have been as parallel among the selection regimes as it could appear on the first glance. First, the statistical interaction between generation and regime that would indicate differential trait changes was not far enough from being significant (p = 0.13 and p = 0.25 for minimum and median generation times, respectively) to be a robust indicator of parallel evolution, in particular when considering the high number of factor levels (the model accounted for the twelve lines within each regime, three regimes, and four generations) with a relatively moderate sample size. Second, the effect size in the fast regime was at least twice as high as in the other two regimes, indicating that in the fast regime the adaptations manifested constantly over the four generations. In contrast, fluctuations in the slow and the control regime were much higher and were at least partly due to random fluctuations (see also Fig. 3).

In any case, we cannot exclude some inadvertent selection leading to shorter generation times in all regimes. For practical reasons we bred mites without beetles, a setting that deviates considerably from the conditions found in nature, where mites may receive timing triggers from the beetles. The beetles also considerably alter the microbiome of the carcass^33–35^, an effect that could slow down the decay of the resource. Assuming the resource decays quicker without the microbial management by beetles, it is possible that the mites were selected for a quicker development when kept in the laboratory, even without any experimental selection. The effect of this type of selection may have already manifested in the first four generations in which we generated a genetically variable stock population without applying any experimental selection. However, the process would have reduced the genetic variability for development time and thus reduced the effect our selection could have produced. Overall, the fact that we still find fast lines to consistently develop quicker than the slow and control lines in the fourth generation suggests that our experimental selection still dominated any potential inadvertently applied selection.

### How is genetic variation maintained?

Life history traits are often less heritable than other traits^2^. Still, there are several reasons for the maintenance of genetic variation, e.g. antagonistic pleiotropy with other traits or gene x environment interactions^36^. Generation time, the trait we examined here, depends much on temperature and faster mites may have a fitness advantage in colder seasons. Patchy environments can maintain genetic variation when selection differs between patches. Indeed we know that beetle brood care differs in length between two German populations^37^. Gene flow between these populations may be sufficient to maintain some variation in both populations. On a smaller scale, the occasional host mismatch may be enough to keep “fast alleles” at sufficient frequencies. After all, the costs of developing too fast appear to be low: Slow development might allow for more food intake during development, resulting in stronger and larger individuals which are better in interference competition (e.g. between males for access to females)^16,38^ and large females may lay more eggs^39^. In our measurements of podonotal and opisthonotal shield size, we did not find clear evidence of fast-selected lines having smaller deuteronymphs. Slow development could also yield more robust individuals, which may increase lifespan^40^ and thus the chance to survive until the carrier beetles find a new carcass. However, these types of benefits of slow development are quantitative and may have less of an impact than the risk of missing the parental beetles, which would immediately reduce fitness to zero for most individuals.

### Potential consequences of adaptation in development time

The standing variation we found in the Freiburg *P. carabi* population suggests that there is some evolutionary potential for dealing with accidental host switches. If these switches were followed by multiple generations of reproduction alongside the novel host species, host-specific sub-populations may form and eventually might become reproductively isolated from the source population^27^. The likelihood of evolving isolation depends on one hand on the probability of encounters with the original host, which is typically rather high and might cause gene flow inhibiting the evolution of an isolated subpopulation^25^. On the other hand, if adaptation in development time would also change other traits that could promote isolation from the source population, such as host choice or mating, reproductive isolation could be quickly established^41^. The mite species differ in size; if there was size-assortative mating in *Poecilochirus*, either by choice or because of incompatibility during copulation, sympatric speciation could occur^19^. Interestingly, we found our selection lines to vary in deuteronymph size independent of selection. When founder populations go through a bottleneck after a host switch, morphological incompatibility might thus quickly evolve. The selection on development time further changed deuteronymph morphology. It is thus possible that selection on development might cause morphological changes also in the adults that reduce the chance of mating with the source population. However, so far there is no evidence for repeated speciation within the same continent in the *P. carabi* species complex^8,22^ (Schedwill & Nehring unpublished data), suggesting that prolonged geographical isolation may be required for speciation to be successful.

## Conclusions

Genetic variation in life history traits is often reduced. In phoretic *Poecilochirus* mites, development time is a life history trait that is crucially adapted to the host beetles’ brood care duration. In a selection experiment we showed that there is still enough standing variation in this trait to allow for rapid adaptation, for example following a host switch. This means that the mites have the evolutionary potential to deal with host switches and adapt to novel environments, which could potentially lead to mite speciation on different hosts.

## Electronic supplementary material

Supplementary Material

Table S1

Table S2

Table S3

## Data Availability

The datasets generated and analysed during the current study are available from Supplementary Tables S1–S3.
