# Supplementary material for: Rapid adaptation in phoretic mite development time
Source: Sci Rep. 2018 Nov 7;8:16460. doi: 10.1038/s41598-018-34798-6 (PMC6220314; doi:10.1038/s41598-018-34798-6)
Supplement: Supplementary file 1 — Supplementary Material [file 41598_2018_34798_MOESM1_ESM.pdf]

Supplementary Material

Supplementary Table S1: *Generation time [in days] over four generations of selection in breeding groups.* We exposed mites to three selection regimes (slow, control, fast), with twelve lines per regime. Each generation, every line was split up into three breeding groups (group_number 1-3). We measured the median generation time (half of the deuteronymphs developed), the day the first and the last deuteronymph developed, and the total number of developed offspring per breeding group (separate csv file).

Supplementary Table S2: *Generation time [in days] when bred in pairs of fourth-generation mites*. Each mite pair was labelled with its selection regime, selection line, and a consecutive number. The dataset contains recordings of the number of days from the copulation of the initial mite pair until the appearance of the first larva, first protonymph, and first deuteronymph, as well as the day 50% of deuteronymphs (median) and all deuteronymphs (last) were developed. The last column contains the total number of offspring that developed into deuteronymphs (separate csv file).

Supplementary Table S3: *Measures of the podonotal and opisthonotal shields of fourth-generation deuteronymphs.* Measures were taken from mites of all three selection regimes (slow, control and fast). Of each selection line ten consecutively numbered individuals were measured. We measured the length [PSL and OSL] (in the middle) and width [PSW and OSW] (at the widest point) of both the podonotal and opistonotal shield (see Fig.6). Values are in µm (separate csv file).

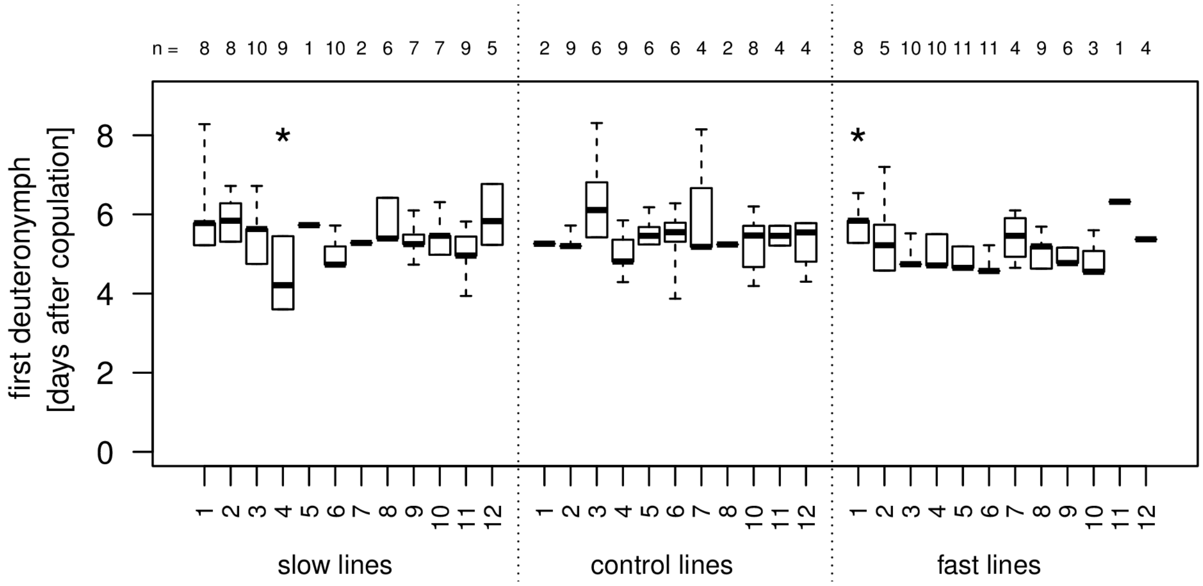

Supplementary Figure S1: Development time for each replicate line of the fourth generation when bred in pairs. Development is measured in days from the first copulation of the parents until the first individual moults into a deuteronymph. Lines marked with * differ from the other lines of the same regime (Tukey test for honest significant differences, $p < 0.05$).

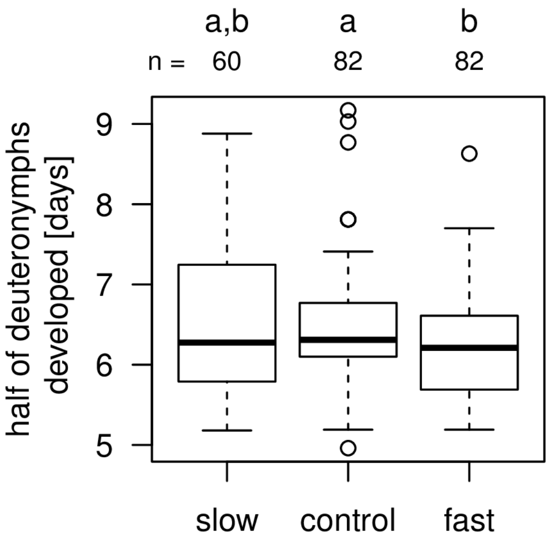

Supplementary Figure S2: Median time for deuteronymph generation time differs between the three selection regimes. Boxplots depict median (thick line), interquartile range (box), minimum and maximum. Numbers are sample sizes; groups with identical letters do not differ from each other in a Tukey post hoc test, $p < 0.05$.

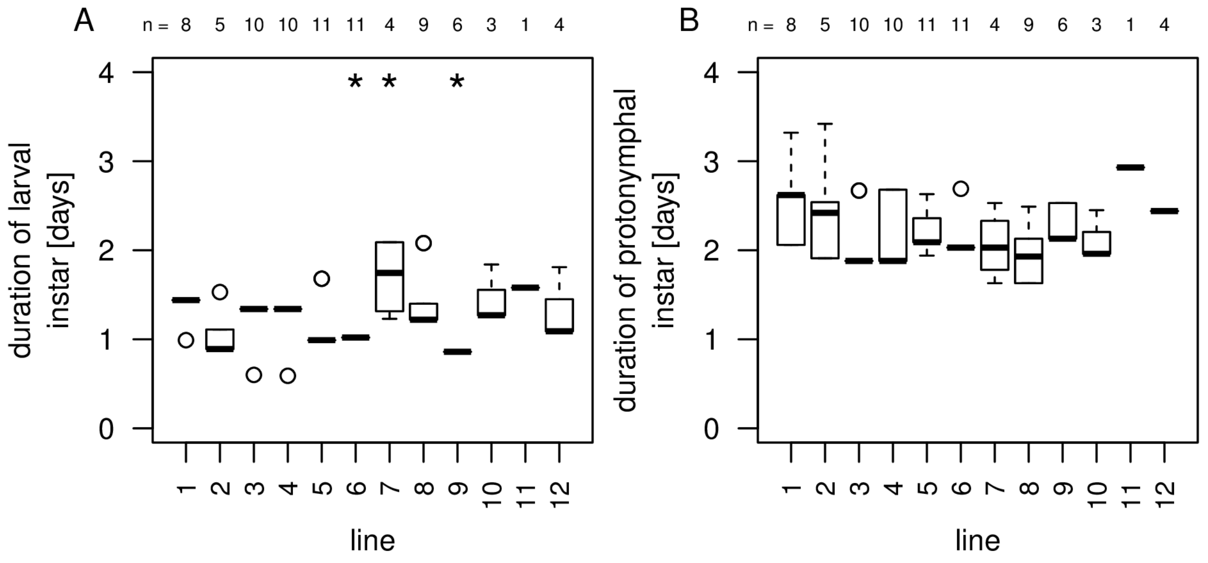

Supplementary Figure S3: Duration of the larval (A) and protonymphal (B) instar after four generations of selection in the fast regime, for each replicate line. Although an ANOVA indicates variation among lines in both stages ($p < 0.001$ and $p < 0.05$, respectively), a Tukey post-hoc test only confirms differences between individual lines for the larval instar (*Tukey post hoc test, $p < 0.05$; line 6 differs from lines 7 and 8; line 7 differs furthermore from lines 2, 5 and 9; and line 9 differs from lines 1 and 8).
